# A phase II randomized trial of RAdium-223 dichloride and SABR Versus SABR for oligomEtastatic prostate caNcerS (RAVENS)

**DOI:** 10.1186/s12885-020-07000-2

**Published:** 2020-06-01

**Authors:** Hamza Hasan, Matthew P. Deek, Ryan Phillips, Robert F. Hobbs, Reem Malek, Noura Radwan, Ana P. Kiess, Shirl Dipasquale, James Huang, Terry Caldwell, Jessica Leitzel, Danielle Wendler, Hao Wang, Elizabeth Thompson, Jonathan Powell, Sara Dudley, Curtiland Deville, Stephen C. Greco, Daniel Y. Song, Theodore L. DeWeese, Michael A. Gorin, Steven P. Rowe, Sam Denmeade, Mark Markowski, Emmanuel S. Antonarakis, Michael A. Carducci, Mario A. Eisenberger, Martin G. Pomper, Kenneth J. Pienta, Channing J. Paller, Phuoc T. Tran

**Affiliations:** 1 Department of Radiation Oncology & Molecular Radiation Sciences, The Sidney Kimmel Comprehensive Cancer Center, Johns Hopkins University School of Medicine, 1550 Orleans Street, CRB2 Rm 406, Baltimore, MD 21231 USA; 2 Department of Medical Oncology, The Sidney Kimmel Comprehensive Cancer Center, Johns Hopkins University School of Medicine, Baltimore, MD USA; 3 The Russell H. Morgan Department of Radiology and Radiological Science, Johns Hopkins University School of Medicine, Baltimore, MD USA; 4 Division of Biostatistics and Bioinformatics, Sidney Kimmel Comprehensive Cancer Center, Johns Hopkins University School of Medicine, Baltimore, MD USA; 5 The James Buchanan Brady Urological Institute and Department of Urology, Johns Hopkins University School of Medicine, Baltimore, MD USA; 6 Cancer Invasion and Metastasis Program, The Sidney Kimmel Comprehensive Cancer Center, Johns Hopkins University School of Medicine, Baltimore, MD USA

**Keywords:** Oligometastatic, Prostate, Cancer, Stereotactic ablative radiation (SABR), Radium-223, Bone, Metastasis

## Abstract

**Background:**

Metastasis directed therapy (MDT) for patients with oligometastatic disease is associated with improvements in progression free survival (PFS) and overall survival (OS) compared to systemic therapy alone. Additionally, within a prostate-cancer-specific cohort, MDT is able to forestall initiation of androgen deprivation therapy (ADT) in men with hormone-sensitive, oligometastatic prostate cancer (HSOPCa) compared to observation. While MDT appears to be safe and effective in HSOPCa, a large percentage of men will eventually have disease recurrence. Patterns of failure in HSOPCa demonstrate patients tend to have recurrence in the bone following MDT, raising the question of sub-clinically-apparent osseous disease. Radium-223 dichloride is a radiopharmaceutical with structural similarity to calcium, allowing it to be taken up by bone where it emits alpha particles, and therefore might have utility in the treatment of micrometastatic osseous disease. Therefore, the primary goal of the phase II RAVENS trial is to evaluate the efficacy of MDT + radium-223 dichloride in prolonging progression free survival in men with HSOPCa.

**Methods:**

Patients with HSOPCa and 3 or less metastases with at least 1 bone metastasis will be randomized 1:1 to stereotactic ablative radiation (SABR, also known as stereotactic body radiation therapy (SBRT)) alone vs SABR + radium-223 dichloride with a minimization algorithm to balance assignment by institution, primary intervention, prior hormonal therapy, and PSA doubling time. SABR is delivered in one to five fractions and patients in the SABR + radium-223 dichloride arm will receive six infusions of radium-223 dichloride at four-week intervals. The primary end point is progression free survival. The secondary clinical endpoints include toxicity and quality of life assessments, local control at 12 months, locoregional progression, time to distant progression, time to new metastasis, and duration of response.

**Discussion:**

The RAVENS trial will be the first described phase II, non-blinded, randomized study to compare SABR +/− radium-223 dichloride in patients with HSOPCa and 3 or less metastases with at least one bone metastasis. The primary hypothesis is that SABR + radium-223 dichloride will increase median progression-free survival from 10 months in the SABR arm to 20 months in the SABR + radium-223 dichloride arm.

**Trial registrations:**

Clinicaltrials.gov. Identifier: NCT04037358. Date of Registration: July 30, 2019. Date of First Participant Enrolled: August 9, 2019. Date of Last Approved Amendment: October 16, 2019. Protocol Version: Version 5.

## Background

Cancer is currently the second leading cause of death annually in the United States, often times due to the development of metastatic disease. Systemic therapies are first line treatments in these instances and they improve survival in patients with metastatic disease. However, they are generally not considered curative for patients with solid metastatic tumors. While systemic therapies improve outcomes, that improvement can come at the expense of side effects that negatively impact patients’ quality of life [1]. Thus, there have been intense efforts to develop novel therapies to improve oncologic outcomes while attempting to balance their toxicity profile.

As the experience treating metastatic disease has evolved, so too has our understanding of the natural history and biology of metastatic cancer. Metastatic disease appears to act along a spectrum that ranges from a single macroscopic metastatic deposit to widespread metastatic disease [2]. Cases with one or a few metastases (typically five or less) have been termed oligometastatic, a state first hypothesized by Hellman and Weichselbaum in the 1990s [2]. The implication of an oligometastatic state is that aggressive metastasis directed therapy (MDT) aimed at all metastatic sites can lead to long term disease control and possibly even a cure [3–6].

The feasibility of MDT has been aided by the development of stereotactic ablative radiation (SABR), a precise form of radiation therapy that allows delivery of high doses of radiation in a small number of treatment sessions. The high lesional control rates seen with SABR, in conjunction with its modest side effect profile, have since resulted in an increasing trend to treat oligometastatic lesions in an attempt to improve overall survival (OS) and progression free survival (PFS), delay initiation of systemic therapies with unfavorable toxicity profiles, and offer treatment breaks for individuals amassing toxicity from systemic therapy [7].

There is now a reasonable body of literature demonstrating the importance of local therapy in patients with oligometastatic disease. Initial evidence came from trials of non-small cell lung cancer (NSCLC) that randomized patients with de novo oligometastatic disease without progression on systemic therapy to continued maintenance systemic therapy/observation or consolidative local therapy and demonstrated improved PFS and OS with local therapy [8, 9]. Further supporting evidence came from the recently published SABR-COMET phase II trial which randomized patients with oligorecurrent disease (and up to five metastatic lesions) with a variety of malignant histologies to receive either standard of care palliative treatments or standard of care palliative treatments plus SABR to all metastatic lesions. The results showed that the median OS in the standard of care palliative treatments arm was 28 months versus 41 months in the standard of care palliative treatments plus SABR arm [10].

Within the prostate cancer literature, numerous retrospective reports have documented the safety and feasibility of using SABR to treat oligometastatic lesions. However, the STOMP trial represented the first reported prospectively randomized trial of MDT for a prostate cancer cohort and investigated the ability of SABR to forestall initiation of androgen deprivation therapy (ADT) in men with HSOPCa with three or fewer detectable metastases. The primary endpoint was ADT-free survival, which was lengthened in men randomized to MDT versus observation (21 vs 13 months) [11, 12]. Our prospective phase II ORIOLE trial, which randomized men with oligometastatic disease to either MDT or observation, also reported an improvement in the primary endpoint of progression adding to the literature surrounding MDT in HSOPCa [13, 14].

While MDT appears to be associated with favorable outcomes in HSOPCa, a large percentage of men will eventually have disease recurrence. Patterns of failure in HSOPCa treated with MDT demonstrate that patients tend to have recurrence in an osseous site following MDT regardless of the site of the initial treated lesion [7, 15]. This raises the question of whether a significant proportion of patients have subclinical micrometastatic disease in bone sites and, if so, how this knowledge might be leveraged to improve outcomes following MDT. Radium-223 dichloride, hereafter referred to as [^223^Ra]RaCl_2_, is a radiopharmaceutical approved by the US FDA for use in the treatment of castration-resistant prostate cancer (CRPC) with osseous metastases [16], may well be suited for this purpose. Radium-223 is effective in this regard due to its structural similarity to calcium, which causes it to be taken up in areas of bone remodeling where it then emits alpha particles [17]. Traditional beta particle-emitting radiopharmaceuticals, especially those with uptake in bone, have bone marrow as a potential organ at risk for toxicity and this was also initially a concern for [^223^Ra]RaCl_2_. However, the short range of alpha particles relative to the size of marrow cavities (19) allows ^223^Ra to treat osseous metastases while sparing normal tissue including bone marrow, leading to minimal side effects [18–20]. The efficacy of [^223^Ra]RaCl_2_ in the treatment of osseous metastases in CRPC was demonstrated in a phase III randomized trial, the ALSYMPCA study, in which 921 patients were randomly assigned in a 2:1 ratio to receive six injections of [^223^Ra]RaCl_2_ every 4 weeks or matching placebo. Those receiving [^223^Ra]RaCl_2_ experienced a survival benefit (median, 14.9 months vs. 11.3 months) and an improvement in quality of life [16]. Therefore, the goal of our phase II RAVENS trial will be to evaluate SABR to all metastatic sites with or without the addition of [^223^Ra]RaCl_2_ in men with HSOPCa and at least one osseous metastasis.

## Methods

### Ethics approval

This study was approved by the Institutional Review Board (IRB) of Johns Hopkins University (IRB00188450). The RAVENS trial is registered at the US National Institutes of Health (ClinicalTrials.gov) #NCT04037358.

### Objectives

- Primary Objective
- To assess progression-free survival of men who have HSOPCa after randomization to SABR versus SABR and [^223^Ra]RaCl_2_.
- Secondary Objectives
- To assess the toxicity of SABR + [^223^Ra]RaCl_2_ in patients with HSOPCa
- To determine local control at 12-months following SABR versus SABR + [^223^Ra]RaCl_2_ in patients with HSOPCa
- To assess time to locoregional progression, time to distant progression, time to new metastasis, and duration of response following SABR versus SABR + [^223^Ra]RaCl_2_.
- To assess ADT-free survival following SABR versus SABR + [^223^Ra]RaCl_2_.
- To assess quality of life following SABR versus SABR + [^223^Ra]RaCl_2_.
- To enumerate circulating tumor cells (CTC) using Epic Sciences’ High Definition Circulating Tumor Cell (HD-CTC) platforms (Epic Sciences, San Diego, CA, USA) at Baseline and Day 181.
- To enumerate circulating tumor DNA (ctDNA) using Cancer Personalized Profiling by deep sequencing (CAPP-Seq) at Baseline, Day 91, Day 181, and Day 361.
- To quantitatively sequence T-Cell receptor (TCR) repertoires using peripheral blood monocytes and the ImmunoSEQ platform (Adaptive Biotechnologies, Seattle, WA, USA) at Baseline and Day 91.
- To evaluate immunophenotypes of peripheral blood mononuclear cells (PBMC)
- To determine the frequency of germline DNA repair mutations in the HSOPCa.

### Inclusion criteria

1. Patients must have at least one, and up to three, asymptomatic metastatic tumor(s) of the bone or soft tissue (with at least one bone metastasis), diagnosed within the past 6-months, that are ≤5.0 cm or < 250 cm^3^
2. Histologic confirmation of prostate cancer (primary or metastatic tumor).
3. Patients must have had their primary tumor treated with surgery and/or radiation.
4. PSA doubling time (PSADT) < 15 months. PSADT will be calculated using as many PSA values that are available from time of relapse (PSA > 0.2). To calculate PSADT, the Memorial Sloan Kettering Cancer Center Prostate Cancer Prediction Tool will be used, which can be found at the following web site: https://www.mskcc.org/nomograms/prostate/psa-doubling-time.
5. Patients may have had prior systemic therapy and/or ADT associated with treatment of their primary prostate cancer. Patients may have had ADT associated with salvage radiation therapy (to the primary prostate cancer or pelvis is allowed).
6. PSA ≥ 0.5 ng/mL but ≤ 50 ng/mL
7. Any testosterone lab within the past 6 months > 50 ng/dL.
8. Patients must be ≥ 18 years of age.
9. Patients must have a life expectancy ≥ 12 months.
10. Patients must have an Eastern Cooperative Oncology Group (ECOG) performance status ≤ 2.
11. Patients must have normal organ and marrow function before the first administration of [^223^Ra]RaCl_2_ defined as: the absolute neutrophil count (ANC) should be ≥ 1.5 × 10^9^/L, the platelet count ≥ 100 × 10^9^/L and hemoglobin ≥ 10 g/dL.
12. Patients must have the ability to understand and the willingness to sign a written informed consent document.
13. Lactate dehydrogenase (LDH) level obtained within 6 months of enrollment.

### Exclusion criteria

1. No more than 3 years of ADT is allowed, with the most recent ADT treatment having occurred greater than 6 months prior to enrollment.
2. Prostate-specific membrane antigen (PSMA)-targeted positron emission tomography (PET)/magnetic resonance imaging (MRI) or PSMA-PET/computed tomography (CT) scan within the past 6 months with results that demonstrate more disease lesions than baseline CT/bone scan.
3. Castration-resistant prostate cancer (CRPC).
4. Spinal cord compression or impending spinal cord compression.
5. Suspected pulmonary and/or liver metastases (greater ≥ 10 mm in largest axis).
6. Patients receiving any other investigational therapeutic agents.
7. Patients receiving abiraterone and prednisone.
8. Patients participating in a concurrent treatment protocol.
9. Serum creatinine > 3 times the upper limit of normal.
10. Total bilirubin > 3 times to upper limit of normal.
11. Liver Transaminases > 5-times the upper limit of normal.
12. Unable to lie flat during or tolerate SABR.
13. Refusal to sign informed consent.

### Evaluation of randomization and blinding

**Fig. 1:**
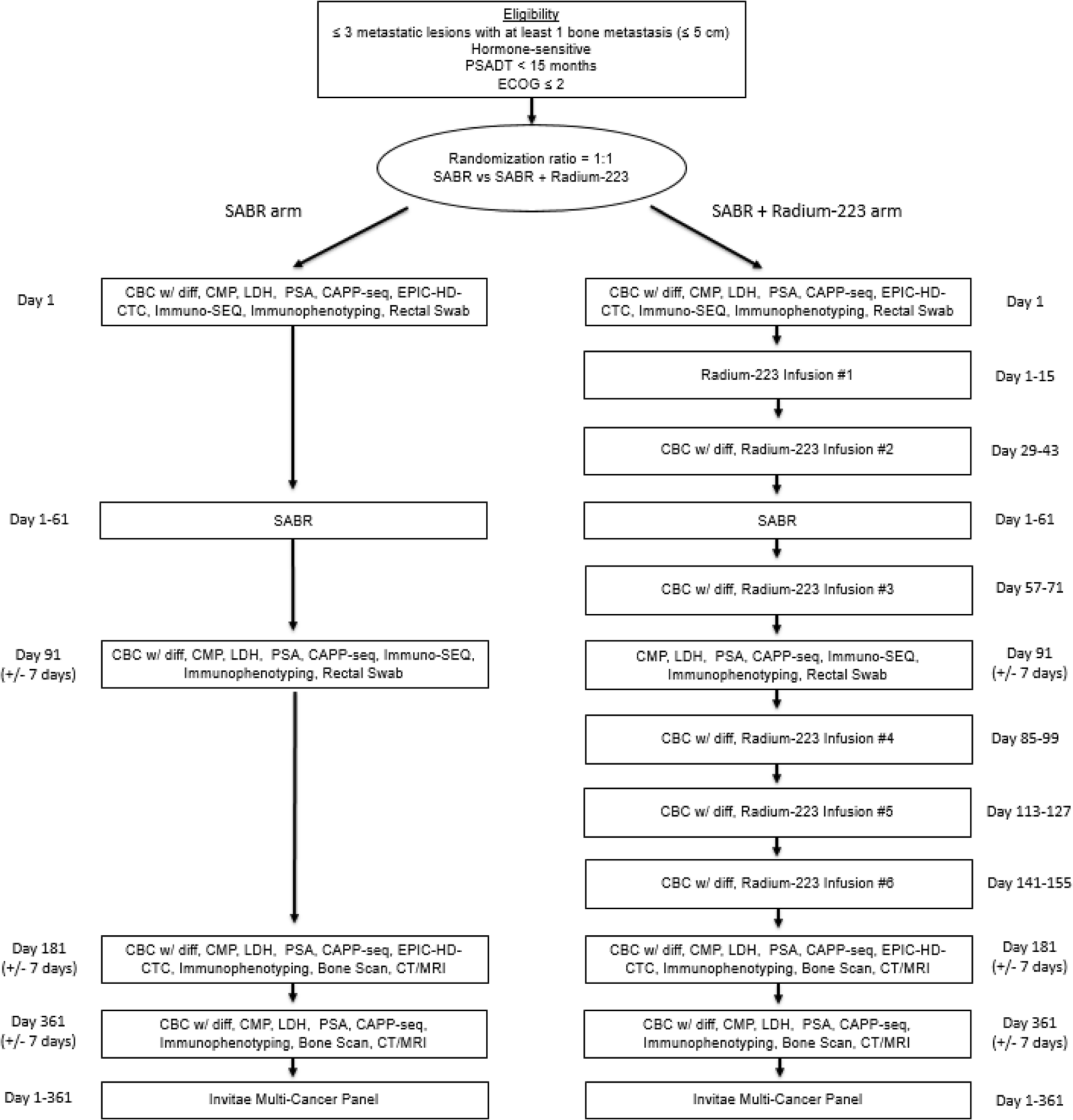
Trial Schema

This study is a multi-site, non-blinded, randomized Phase II trial in patients with oligometastatic prostate cancer with three or less metastases and at least one bone metastasis. Eligible patients will be randomized at a 1:1 ratio to one of the treatment arms: the SABR arm or the SABR + [^223^Ra]RaCl_2_ arm (Fig. 1). The study coordinator will use an interactive web response system (IWRS) to randomize each patient. The randomization will be performed using a minimization algorithm [21] that utilizes the following stratification factors: Initial treatment (Surgery or Radiation), Prior Hormone Therapy (Yes or No), and PSADT (< 6 months vs 6–14.9 months). The minimization algorithm uses an 85% probability of study arm assignment and has the maximum imbalance set to 4. The randomization will not be blinded and the on-study date for protocol entry will be the day that the study subject is randomized.

### Interventions

The procedures performed during the patients’ pre-cycle visit will be used to determine eligibility by screening patients using the inclusion and exclusion criteria (see Tables 1 and 2).

**Table 1:**
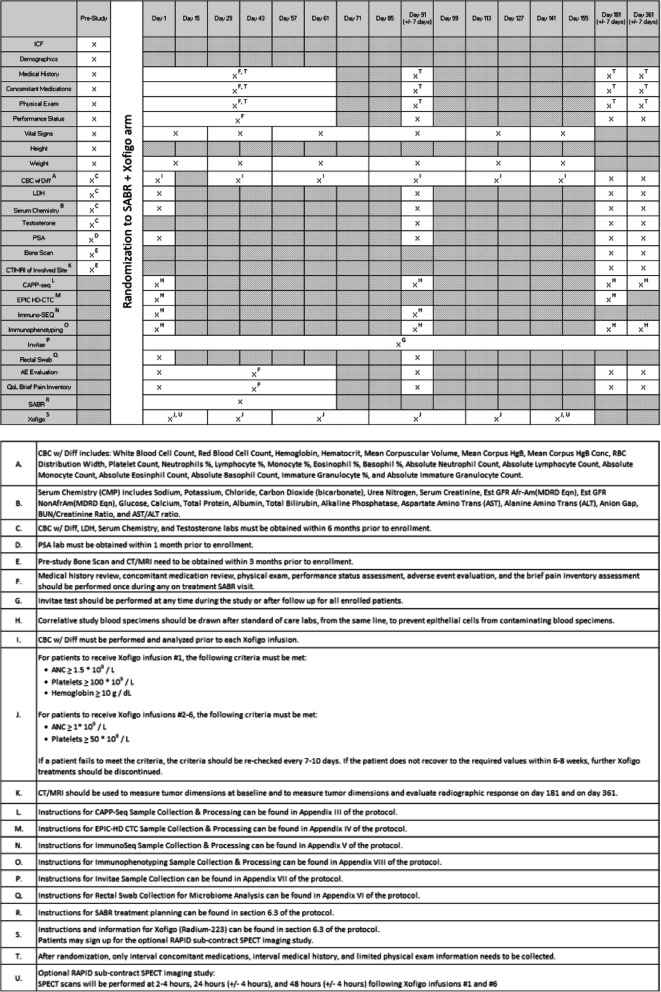
Study Calendar (SABR + Xofigo arm)

**Table 2:**
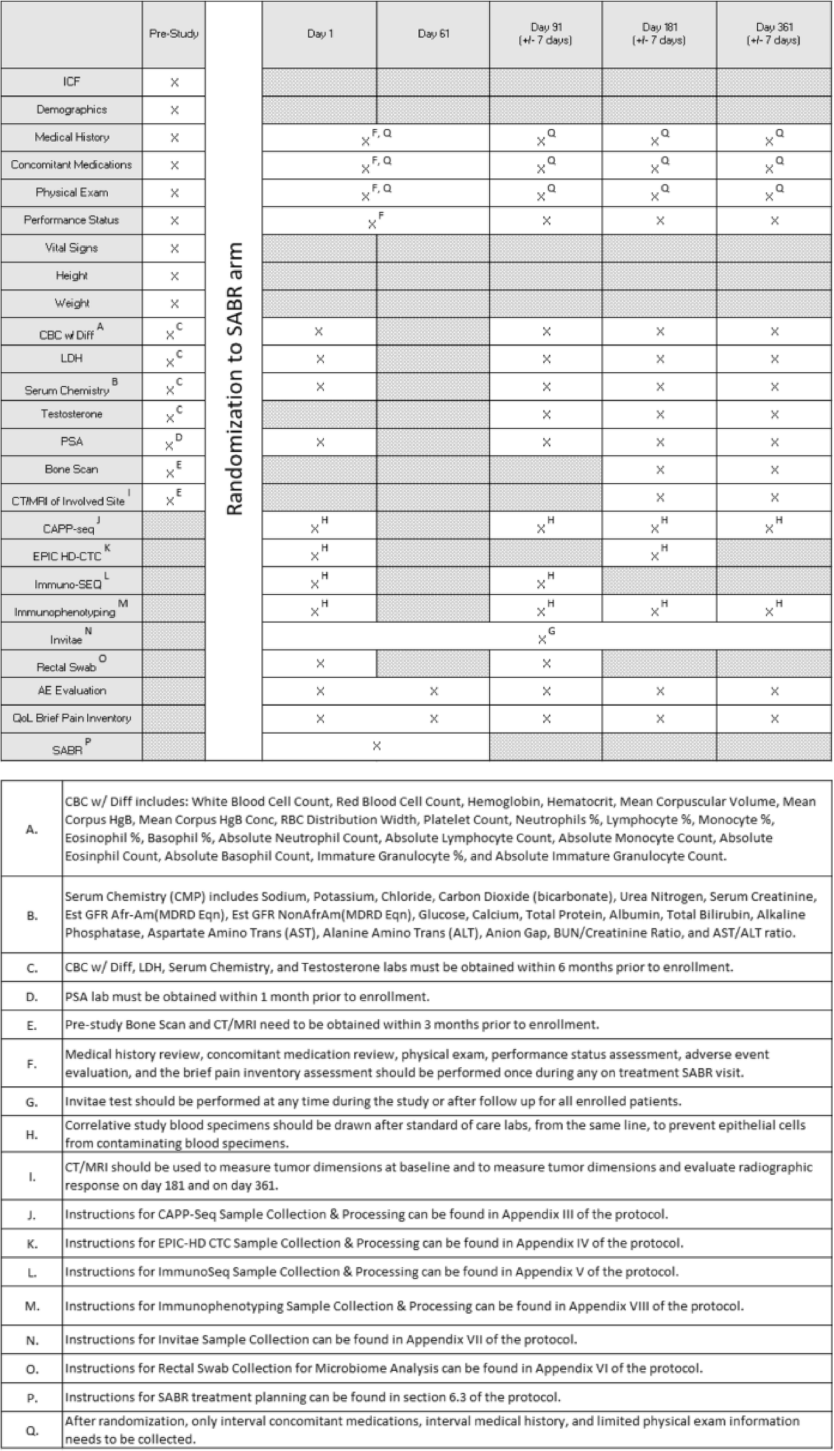
Study Calendar (SABR arm)

The following will need to be completed prior to enrollment:

- PSA level will be obtained within 30 days prior to enrollment.
- Bone scan and CT of the abdomen and pelvis or MRI of the pelvis will be obtained within 3 months prior to enrollment.
- Complete blood count (CBC) with differential, LDH, serum chemistry, and testosterone will be obtained within 6 months prior to enrollment.
- Demographics, medical history, concomitant medications, physical exam, performance status, height and weight will be obtained within 30 days prior to enrollment.

The following will need to be completed on Day 1:

- Vital signs and weight.
- LDH, serum chemistry, PSA, CAPP-seq, EPIC-HD-CTC, Immuno-SEQ, immunophenotyping, and rectal swab.
- CBC with differential (prior to [^223^Ra]RaCl_2_ infusion, patients must meet ANC ≥ 1.5 * 10^9^/L and platelets ≥ 100 * 10^9^/L)
- Brief pain inventory (short form) and common terminology criteria for adverse events (CTCAE) v4 adverse event evaluation.

The following will need to be completed between Day 1 and Day 15 (SABR + [^223^Ra]RaCl_2_ arm only):

- Vital signs and weight.

The following will need to be completed between Day 1 and Day 61, during the patient’s SABR visit:

- Interval medical history, interval concomitant medications, limited physical exam, performance status, CTCAE v4 adverse event evaluation, and the Brief Pain Inventory (short form).

The following will need to be completed between Day 1 and Day 361:

- Invitae Multi-Cancer Panel

The following will need to be completed between Day 29 and Day 43 (SABR + [^223^Ra]RaCl_2_ arm only)

- Vital signs and weight.
- CBC with differential (prior to [^223^Ra]RaCl_2_ infusion, patients must meet ANC ≥ 1 * 10^9^/L and platelets ≥ 50 * 10^9^/L)

The following will need to be completed between Day 57 and Day 71 (SABR + [^223^Ra]RaCl_2_ arm only)

- Vital signs and weight.
- CBC with differential (prior to [^223^Ra]RaCl_2_ infusion, patients must meet ANC ≥ 1 * 10^9^ / L and platelets ≥ 50 * 10^9^ / L)

The following will need to be completed between Day 85 and Day 99 (SABR + [^223^Ra]RaCl_2_ arm only)

- Vital signs and weight.
- CBC with differential (prior to [^223^Ra]RaCl_2_ infusion, patients must meet ANC ≥ 1 * 10^9^ / L and platelets ≥ 50 * 10^9^ / L)

The following will need to be completed on Day 91:

- Interval medical history, interval concomitant medications, limited physical exam, performance status, CTCAE v4 adverse event evaluation, and the Brief Pain Inventory (short form).
- CBC with differential, LDH, serum chemistry, testosterone, PSA, CAPP-seq, Immuno-SEQ, immunophenotyping, and rectal swab.

The following will need to be completed between Day 113 and Day 127 (SABR + [^223^Ra]RaCl_2_ arm only)

- Vital signs and weight.
- CBC with differential (prior to [^223^Ra]RaCl_2_ infusion, patients must meet ANC ≥ 1 * 10^9^ / L and platelets ≥ 50 * 10^9^ / L)

The following will need to be completed between Day 141 and Day 155 (SABR + [^223^Ra]RaCl_2_ arm only)

- Vital signs and weight.
- CBC with differential (prior to [^223^Ra]RaCl_2_ infusion, patients must meet ANC ≥ 1 * 10^9^ / L and platelets ≥ 50 * 10^9^ / L)

The following will need to be completed on Day 181:

- Interval medical history, interval concomitant medications, limited physical exam, performance status, CTCAE v4 adverse event evaluation, and the Brief Pain Inventory (short form).
- CBC with differential, LDH, serum chemistry, testosterone, PSA, CAPP-seq, immunophenotyping, and EPIC HD-CTC.
- Bone scan and CT of the abdomen and pelvis or MRI of the pelvis..

The following will need to be completed on Day 361:

- Interval medical history, interval concomitant medications, limited physical exam, performance status, CTCAE v4 adverse event evaluation, and the Brief Pain Inventory (short form).
- CBC with differential, LDH, serum chemistry, testosterone, PSA, CAPP-seq, and immunophenotyping.
- Bone Scan and CT of the abdomen and pelvis or MRI of the pelvis.

### Radiation planning and dosage

- SABR Treatment Planning

CT- and/or MRI-simulation will be performed with fabrication of a radiation therapy immobilization device (such as the Alpha Cradle) which will be custom made for each patient. The treating radiation oncologist will identify the location of the tumor. Gross tumor volume (GTV) delineation will be performed with a diagnostic radiologist on sequential axial computed tomography images. A radiosurgical treatment plan will be developed based on tumor geometry and location. The clinical tumor volume (CTV) will equal the GTV. The dose will be prescribed to the minimal isodose line that completely covers the planning target volume (PTV) which is equal to CTV plus a 3–5 mm margin). Adjacent normal structures including but not limited to the heart, esophagus, aorta, spinal cord, kidneys, rectum, bowel, liver, and stomach within 5 cm of the CTV will be identified for the purpose of limiting incidental radiation to these structures.

In addition, prior to treatment delivery, a four-dimensional cone beam CT study will be performed on individual patients to assess respiration in these patients and to determine tumor targeting accuracy for those tumors that may be subject to respiratory motion such as those in the bones of the thorax. If tumor motion is greater than 5 mm, PTV will be expanded to account for respiration.

SABR will be delivered in 1 to 5 fractions, and the dose and fractionation schedule will depend on the size and location of the lesion and the surrounding normal tissue constraints in accordance with AAPM Task Group 101 recommendations. Typical doses include 16–24 Gy in 1 fraction, 48–50 Gy in 4 fractions, and 40–60 Gy in 5 fractions.

### NIST standardization update

The radioactivity quantification of ^223^Ra in [^223^Ra]RaCl_2_ is based on the US National Institute of Standards and Technology’s (NIST) primary standardization. The US NIST prepares the standard reference material (SRM) using an official dial setting (primary standardization) as published [22]. The NIST SRM is used to calibrate the instruments in production and quality control for both the drug substance and drug product. Additionally, the NIST SRM is used to prepare the NIST traceable ^223^Ra reference materials which are then sent to the end-users (e.g., nuclear medicine laboratory physicians or technicians) for dial-setting of their dose calibrators, to allow verification of the patient dose.

In 2014, NIST performed a re-assessment of the primary standardization based on preliminary information suggesting a potential discrepancy of approximately 8–10% between the published NIST primary standardization [22] and results obtained by other national metrology institutes (United Kingdom, Germany, Japan). After completion of the re-assessment, NIST reported their findings [23] and had issued a revised NIST SRM in 2015. The discrepancy in the NIST standardization was determined to be − 9.5% between activity values obtained using the old reference standard relative to the new primary standardization. Consequently, the current numerical values have been updated by approximately + 10.5%.

### Radium-223 dichloride administration

Dosage Forms and Strength:

[^223^Ra]RaCl_2_ is available in single-use vials containing 6 mL of solution at a concentration of 1100 kBq/mL (29.7 μCi/mL) (previously valued at 1000 kBq/mL (27.0 μCi/mL) before implementation of the NIST quantification update) at the reference date with a total activity of 6600 kBq/vial (178 μCi/vial) (previously valued at 6000 kBq/vial (162 μCi/vial) before implementation of the NIST quantification update) at the reference date.

Before the first administration of [^223^Ra]RaCl_2_, the ANC will be ≥1.5 × 10^9^/L, the platelet count ≥100 × 10^9^/L and hemoglobin ≥10 g/dL. Before subsequent administrations of [^223^Ra]RaCl_2_, the ANC should be ≥1 × 10^9^/L and the platelet count ≥50 × 10^9^/L. If there is no recovery to these values within 6 to 8 weeks after the last administration of [^223^Ra]RaCl_2_, despite receiving supportive care, further treatment with [^223^Ra]RaCl_2_ will be discontinued. Patients with evidence of compromised bone marrow reserve will be monitored closely and provided with supportive care measures when clinically indicated. [^223^Ra]RaCl_2_ will be discontinued in patients who experience life-threatening complications despite supportive care for bone marrow failure.

The dose regimen of [^223^Ra]RaCl_2_ is 55 kBq (1.49 μCi) (previously valued at 50 kBq (1.35 μCi) before implementation of the NIST quantification update) per kg body weight, given at 4-week intervals for 6 injections [24].

### Early stopping guidelines

This study will monitor site-specific grade 4/5 toxicity in the SABR + [^223^Ra]RaCl_2_ arm. If it becomes evident that the proportion of grade 4/5 toxicity at specific sites convincingly exceeds 20%, the study will be halted for a safety consultation [14]. Patients with disease progression are individually taken off-study and adverse events are recorded for these patients.

### Statistical analysis

#### Response criteria

Response criteria to treatment will be defined as follows:

- Evaluation of target lesions/PSA response [25]
- Complete Response (CR): Disappearance of all target lesions on a CT scan and bone scan and PSA < pre-SABR PSA
- Partial Response (PR): At least 30% decrease in the sum of the longest diameter (LD) of target lesions, taking as reference the baseline sum LD. Or a third of the lesions are negative or no change by bone scan and PSA ≤ pre-SABR PSA.
- Progressive Disease (PD): At least a 20% increase in the sum of the LD of target lesions, taking as reference the smallest sum LD recorded since the treatment started or the appearance of ≥ 1 new lesion(s) on CT or MRI. Or ≥ 1 new lesion(s) appear by bone scan. Or ≥ 25% increase in PSA from nadir or > 50 ng/mL.
- Stable Disease (SD): Neither sufficient shrinkage to qualify for PR nor sufficient increase to qualify for PD, taking as reference the smallest sum LD since the treatment started. Or PSA ≥ pre-SABR PSA, but not ≥ 25% increase in PSA from nadir and < 50 ng/mL.

- Evaluation of Best Overall Response
  The best overall response will be the best response recorded from the start of the treatment until disease progression/recurrence (taking as reference for progressive disease the smallest measurements recorded since the treatment started). Best overall response will be based on the overall response of the target lesions.

- Duration of Response
  Response will be defined as evidence of CR, PR, or stable disease. The duration of response will be measured from the start of treatment until the criteria for progression are met.

Duration of CR or PR: The duration of CR or PR will be recorded from the time measurement criteria are met for CR or PR (whichever is first recorded) until the first date that current or progressive disease is objectively documented (taking as reference for progressive disease the smallest measurements recorded since the treatment started).

Duration of Stable Disease: Stable disease is measured from the start of the treatment until the criteria for progression are met, taking as reference the smallest measurements recorded since the treatment started.

- Clinical Response Parameters
- Progression is a composite endpoint defined from the Prostate Cancer Working Group 2 (PCWG2) criteria for metastatic castrate resistant prostate cancer (mCRPC) [26] and our previous trials in a population of men with biochemical failure without metastases [27–29]. Progression will be defined as either: 1) a ≥ 25% increase in PSA from nadir (and by ≥2 ng/mL), requiring confirmation ≥4 weeks later (PCWG2 criteria); and/or, 2) clinical/radiographic-progression defined as symptomatic progression (worsening disease-related symptoms or new cancer-related complications), or radiologic progression (on CT/MRI scan: ≥20% enlargement in sum diameter of soft-tissue target lesions [RECIST 1.1 criteria] [25]; on bone scan: ≥ 1 new bone lesions), initiation of ADT or death due to any cause, whichever occurs first. Death will be considered a severe adverse event here.

- Progression Free Survival (PFS) is defined as the time from starting treatment to the time of progression as defined above. Subjects who do not progress will be censored at the time of the last contact.

- ADT Free Survival (ADT-FS) is defined as the time from starting treatment to the time of initiation of palliative ADT. ADT will typically be initiated on tumor progression and/or development of new metastases. Subjects who do not start ADT will be censored at the time of the last contact.

- Time to Progression (TTP) is defined as the time from starting treatment to the time of first documented tumor progression or new lesions by CT/MRI and/or bone scan or initiation of ADT. Subjects who do not progress will be censored at the time of the last contact. In addition, death from any cause will also be censored.

- Time to New Metastasis (TTNM) is defined as the time from starting treatment to the time of a new documented tumor metastasis by CT/MRI and/or bone scan. Subjects who do not progress will be censored at the time of the last contact.

- Overall Survival (OS) is defined as the time from starting treatment until death due to any cause. For subjects who do not die, time to death will be censored at the time of last contact.

- Locoregional Control (LRC) is defined as the time from starting treatment until local and/or regional relapse is documented

### Statistical analysis

- Analysis of Primary Objective
  This is a randomized, Phase II trial of SABR versus SABR + [^223^Ra]RaCl_2_ in HSOPCa patients. The minimization approach [21] will be applied to ensure balanced assignment to each treatment arm by stratification factors: 1) Initial treatment with surgery vs. radiation therapy; 2) Prior hormonal therapy vs. no prior hormonal therapy; and 3) PSADT < 6 months vs. 6–14.9 months. Baseline PSA level is defined as that measured Day 1 following randomization.
  The primary outcome of interest is PFS, defined as the time from the date of randomization to the date of disease progression or death, whichever happens earlier. For those who are alive and do not have progressive disease, PFS will be censored at the time of the last scan. The Kaplan-Meier method will be used to summarize PFS and log-rank test will be used to compare PFS between the two arms. The analysis population includes all randomized subjects based on the intent-to-treat principle. Those who are lost to follow up will be censored in the analysis.
- Analysis of Secondary Objectives

Secondary objectives will be analyzed as follows:

- For safety analysis, adverse events will be summarized by type and grade.
- Kaplan-Meier (KM) estimates will be used to summarize ADT-free survival (ADT-FS), time to locoregional progression (TTLP), time to distant progression (TTDP), time to new metastasis (TTNM) and duration of response over time. The median PFS, ADT-FS, TTLP, TTDP, TTNM, and duration of response will be reported.
- The efficacy of SABR + [^223^Ra]RaCl_2_ in men with HSOPCa will also be determined by measuring local control of each lesion at 12-months.
- Quality of life will be assessed using the Brief Pain Inventory form. An overall score will be calculated pre-treatment and at the time of the 2nd radiologic reassessment. The change in score will be evaluated with a paired t-test.

- Sample Size
  The primary endpoint will be PFS. Data from STOMP [12] on this patient population indicate that > 50–60% would show progression as defined above within a 12-month period from SABR, and a median PFS of approximately 10 months. We hypothesize that the addition of [^223^Ra]RaCl_2_ will be able to reduce the risk of progression by 50%. A sample size using a 1:1 randomization scheme of 30 patients per arm will provide 80% power to detect an increase of median PFS from 10 months to 20 months (corresponding to hazard ratio 0.5) with type I error = 0.1, using a one-sided log-rank test. The calculation assumes 18 months of accrual time with an additional follow-up of 12 months after the last patient is randomized. To account for 5% early drop out, we will randomize a total of 64 patients (32 per arm).

## Discussion

Historically, aggressive local therapy has not been used in the management of patients with metastatic disease. However, with improvements in local and systemic therapy options there has recently been great interest in integrating local therapies into the management of patients with metastatic disease. This is especially the case in patients with “oligo,” or few sites of metastases, who may benefit with aggressive consolidation of all macroscopic disease [7, 12, 30–42].

Several studies have now shown the clinical safety and oncologic efficacy of MDT through improvements of PFS and OS in individuals with oligometastatic disease [8–10]. Currently, the definition of oligometastatic revolves around numerical definitions due to its association with outcomes, and therefore most studies have included patients with up to 3–5 metastatic foci [43]. Little evidence exists as to whether local therapy to metastatic lesions benefits patients with higher metastatic burden. The now accruing phase III trial SABR COMET 10 (NCT03721341) is enrolling patients with 4–10 metastatic lesions with a primary end point of OS and will help answer this question [44]. If a benefit to consolidative therapy is noted with higher volume disease, [^223^Ra]RaCl_2_ might be suited for integration into the treatment of these patients given its systemic distribution. The ALSYMPCA trial, which enrolled men with metastatic CRPC, randomized men to [^223^Ra]RaCl_2_ or placebo and demonstrated a survival benefit for those treated with [^223^Ra]RaCl_2_. Of these patients, 85% had > 6 bone lesions at treatment, so [^223^Ra]RaCl_2_ is capable of treating high volume disease [16]. Therefore, it is hoped that the results of RAVENS and SABR COMET 10 will help to inform future management in this cohort of patients.

The hormone dependent nature of prostate cancer allows for the addition of systemic therapies such as ADT to be used in the management of metastatic disease, and ADT is the first line standard of care. However, ADT is associated with side effects causing decrements in quality of life [1], so there is interest in using MDT to forestall initiation of ADT in hormone-sensitive disease. The STOMP trial, which was the first prospectively reported trial studying the efficacy of MDT in forestalling ADT initiation, showed ADT-free survival was lengthened in men randomized to MDT versus observation (21 vs 13 months) without an accompanying decrement in quality of life [12]. The RAVENS trial represents an attempt to intensify therapy in this cohort of patients while still avoiding the unfavorable toxicity profile of ADT. The short range of alpha particles emitted from ^223^Ra results in minimal toxicity outside of occasional bone marrow suppression and thus its combination with SABR should theoretically not result in a large decrement in quality of life over SABR alone as the toxicities are orthogonal. RAVENS attempts to intensify therapy while also considering patterns of failure in oligometastatic prostate cancer following MDT, which demonstrate a trend to failure in the bone regardless of initial treatment location [7, 15]. ^223^Ra is an alpha-emitting radioisotope that is a bone-seeking calcium mimetic and selectively targets areas with increased bone turnover, especially within the microenvironment of osteoblastic or sclerotic metastases [17]. This could make [^223^Ra]RaCl_2_ a powerful tool in forestalling disease recurrence given reported patterns of failure. Several other methods of treatment intensification are currently being investigated within the oligometastatic PCa realm, the most logical of which would be to add additional systemic therapies to MDT. Evidence suggests that the addition of a course of ADT to SABR is associated with promising outcomes. For example, a cohort of 28 men with HSOPCa at Johns Hopkins Hospital treated with a median of 4.3 months of ADT after MDT experienced a 24-month biochemical PFS of 77%, with only 18% of men having restarted ADT at that time [7]. Several prospective trials are thus aiming to combine MDT with systemic agents, including combining SABR with traditional luteinizing hormone releasing hormone agonists/antagonists (NCT03940235), abiraterone (NCT03449719), ipilimumab (NCT03477864), and durvalumab (NCT03795207). Other areas of interest include the optimal radiation volume following nodal recurrence, being studied in the PEACE V – STORM trial, which randomizes patients to MDT and ADT +/− whole pelvis RT (NCT03569241) and the GAP6 initiative which aims to better understand molecular features of oligometastatic prostate cancer.

## Conclusions

The clinical results of MDT in HSOPCa are promising but would benefit from continued novel therapeutic strategies to continue to improve outcomes. Therefore, the RAVENS trial aims to compare MDT alone to MDT plus [^223^Ra]RaCl_2_ for patients with oligometastatic prostate cancer with 3 or less metastases and at least one bone metastasis with the primary goal of achieving improved PFS to 20 months for the patients in the SABR + [^223^Ra]RaCl_2_.

### Data monitoring

A Data Monitoring Committee is in place to monitor the trial. Data and safety monitoring oversight is conducted by the Sidney Kimmel Comprehensive Cancer Center (SKCCC) at Johns Hopkins Safety Monitoring Committee. Per the SKCCC at Johns Hopkins Safety Monitoring plan, the CRO AQ will forward summaries of all monitoring reports to the Safety Monitoring Committee for review.

## Data Availability

Raw data is not currently available for publication as the trial is still accruing patients and has not undergone interim analysis.

## Abbreviations

[223Ra]RaCl2: Radium- 223 Dichloride
223Ra: Radium-223
AAPM: American Association of Physicists in Medicine
ADT: Androgen Deprivation Therapy
ADT-FS: Androgen Deprivation Therapy Free Survival
ANC: Absolute Neutrophil Count
CAPP-Seq: Cancer Personalized Profiling by Deep Sequencing
CBC: Complete Blood Count
CRPC: Castrate Resistant Prostate Cancer
CT: Computed Tomography
CTC: Circulating Tumor Cells
CTCAE: Common Terminology Criteria For Adverse Events
ctDNA: Circulating Tumor DNA
CTV: Clinical Tumor Volume
DNA: Deoxyribonucleic Acid
ECOG: Eastern Cooperative Oncology Group
GAP6: Global Action Plan 6
GTV: Gross Tumor Volume
HD: CTC High Definition Circulating Tumor Cell
HSOPCa: Hormone Sensitive Oligometastatic Prostate Cancer
IRB: Institutional Review Board
IWRS: Interactive Web Response System
KM: Kaplan- Meier
LD: Longest Diameter
LDH: Lactate Dehydrogenase
LRC: Locoregional Control
mCRPC: Metastatic Castrate Resistant Prostate Cancer
MDT: Metastasis Directed Therapy
MRI: Magnetic Resonance Imaging
NIST: National Institute of Standards and Technology
NSCLC: Non-Small Cell Lung Cancer
OS: Overall Survival
PBMC: Peripheral Blood Mononuclear Cells
PCa: Prostate Cancer
PCWG2: Prostate Cancer Working Group 2
PD: Progressive Disease
PET: Positron Emission Tomography
PFS: Progression Free Survival
PR: Partial Response
PSA: Prostate Specific Antigen
PSADT: Prostate Specific Antigen Doubling Time
PSMA: Prostate-Specific Membrane Antigen
PTV: Planned Target Volume
RECIST: Response Evaluation Criteria in Solid Tumors
RT: Radiation Therapy
SABR: Stereotactic Ablative Radiation
SBRT: Stereotactic Body Radiation Therapy
SD: Stable Disease
SRM: Standard Reference Material
TCR: T-Cell Receptor
TTDP: Time to Distant Progression
TTLP: Time to Locoregional Progression
TTNM: Time to New Metastasis
TTP: Time to Progression
US FDA: United States Food and Drug Administration
